# Integrating protein-protein interactions and text mining for protein function prediction

**DOI:** 10.1186/1471-2105-9-S8-S2

**Published:** 2008-07-22

**Authors:** Samira Jaeger, Sylvain Gaudan, Ulf Leser, Dietrich Rebholz-Schuhmann

**Affiliations:** 1 Knowledge Management in Bioinformatics, Humboldt-University Berlin, Unter den Linden 6, 10099 Berlin, Germany; 2 European Bioinformatics Institute, Wellcome Trust Genome Campus, Hinxton, Cambridge, CB10 1SD, UK

## Abstract

**Background:**

Functional annotation of proteins remains a challenging task. Currently the scientific literature serves as the main source for yet uncurated functional annotations, but curation work is slow and expensive. Automatic techniques that support this work are still lacking reliability. We developed a method to identify conserved protein interaction graphs and to predict missing protein functions from orthologs in these graphs. To enhance the precision of the results, we furthermore implemented a procedure that validates all predictions based on findings reported in the literature.

**Results:**

Using this procedure, more than 80% of the GO annotations for proteins with highly conserved orthologs that are available in UniProtKb/Swiss-Prot could be verified automatically. For a subset of proteins we predicted new GO annotations that were not available in UniProtKb/Swiss-Prot. All predictions were correct (100% precision) according to the verifications from a trained curator.

**Conclusion:**

Our method of integrating CCSs and literature mining is thus a highly reliable approach to predict GO annotations for weakly characterized proteins with orthologs.

## Background

Elucidating protein functions is a challenging task and essential to better understand biological processes, cellular mechanisms, evolutionary changes and the onset of diseases. A special electronic resource has been created to capture knowledge on protein function in bioinformatics data resources: the Gene Ontology (GO) [1,2]. The annotation of proteins with GO terms is an ongoing work which is carried out by professional database curators based on literature information and thus tends to be time-consuming. Accordingly, only a relatively small number of proteins has yet been annotated, covering less than 3% of UniProtKb [3]. To increase coverage and to speed up the annotation process, new techniques are required that support curators, for instance by providing them with automatically generated annotation suggestions. However, these suggestions are only helpful if they exhibit very high quality and are rarely wrong.

There are numerous ways to predict protein function [4]. Predictions can be based on the analysis of protein sequences and 3D-structures [5], on orthology relationships, on domain structures, or on the position of proteins within their networks of interacting partners [6,7]. However, many of these methods when used in isolation do not reach the level of precision that is considered as helpful by database curators. A promising way to improve precision (though usually at the cost of recall) is to combine different methods. In particular, text mining could be helpful for generating or validating predictions [8,9]. For instance, Shatkay *et al*. introduced a comprehensive system for predicting the cellular location of eukaryotic proteins by integrating several types of sequence-derived and text-based features [10]. Overall they were able to predict protein localizations with an accuracy of 71% achieving a significant improvement to earlier prediction methods.

Different text mining approaches have been proposed to automatically find GO terms in the literature, e.g., GoPubMed [11] and EBIMed [12]. Using these approaches for function prediction typically results in predictions with less than 35% precision [13]. The main reason accounting for this low precision is that most sentences contain several proteins and GO terms, and thus highly discriminative natural language processing would be needed to detect true associations. Furthermore, it is still difficult to identify GO terms in text in first place which also results in low recall. A number of reasons explain this. First, the GO terminology has not been designed for text mining and does not necessarily mimic the language used in the scientific literature. Second, there is yet little motivation for authors to comply to GO terminology in their publications. Third, natural language is constantly evolving and offers ways to sub-specify existing terminology according to individual needs. Last, authors tend to hedge their statements leading to convoluted expressions [14]. In any case, methods with such a level of precision do not efficiently support the annotation process.

In this paper, we present a novel approach to precisely predict GO terms for poorly annotated proteins. Our approach combines two prediction methods that use complementary data from different resources. In the initial step we generated conserved subgraphs from protein-protein-interaction (PPI) networks from various species. From these subgraphs GO annotations for proteins are predicted, if orthologous proteins with known molecular function are available [15]. These hypothetical new annotations are validated against annotations that are automatically extracted from the scientific literature. Our combined approach for predicting GO terms achieves on automatically preselected proteins a very promising precision (actually 100% for all proteins we probed for), and thus efficiently supports GO curation. Our approach also shows that scientific literature contains relevant information that is complementary to the rich and valuable information contained in public databases.

## Methods

The main components of our approach are the selection of proteins from topologically conserved subgraphs in protein-protein interaction networks and the identification of GO annotation in the scientific literature. Both methods are described in the following sections.

### Predicting protein functions

In [15], we reported on a system which exploits conservation of PPIs for function prediction. For completeness, we briefly describe this method here; see [15] for details.

We generated protein-protein-interaction graphs for *H. sapiens*, *M. musculus*, *D. melanogaster *and *S. cerevisiae *from the Database of Interacting Proteins [16], BIND [17], Mammalian MIPS [18], IntAct [19] and the Human Protein Reference Database [20]. We chose yeast and fly as one of the best studied model organisms in several research fields to compare them on the level of PPIs with human and mouse which hardly have been analysed so far.

**Figure 1:**
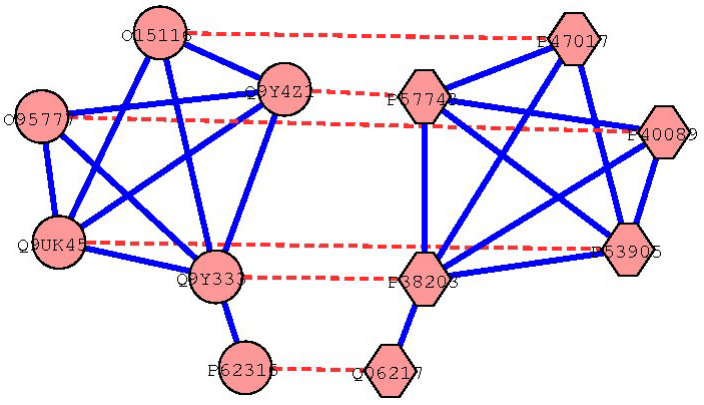
**Example CCS detected between *H. sapiens* and* S. cerevisiae***. The figure shows a conserved and connected subgraph between *H. sapiens *(circle) and *S. cerevisiae *(hexagon). Proteins of both species are involved in mRNA splicing and are known to exhibit splicing factor activity to bind the mRNA and support the splicing process. (Solid lines represent conserved PPIs within a species and dashed lines indicate orthology relationships between proteins.)

We first computed sets of orthologous proteins from different species based on sequence similarity. Subsequently, we detected conserved interactions (interologs [21]) in the interactions graphs of all considered species between those orthologs. Next, we identified maximally connected subgraphs in different species that are topologically isomorph by assembling interologs. Such subgraphs are called conserved and connected subgraphs (CCSs). A CCS (see Figure 1) is defined by a set of orthologous proteins detected in each species and their set of interactions that have been identified in all of the considered species. Proteins in a CCS are associated with available functional annotation obtained from Swiss-Prot [22]. We used a GO-based scoring scheme [23] to measure functional similarity of CCSs to assess how far structural homogeneity given by the CCSs correlates with functional similarity of proteins in the CCSs.

Functional similarity of a CCS can be inferred from proteins contained in a CCS under the following three assumptions. (1) Proteins in the CCS have orthologous proteins in different species. Orthologous proteins evolved from a common ancestor protein and therefore are most likely to have the same function. (2) Orthologous proteins in the CCS interact with again orthologous proteins ("orthologous interactions" – interologs). Proteins interacting with each other usually are involved in similar processes and functions and it has been shown that 70–80% of proteins share at least one function with its interaction partner [24]. (3) Proteins and their interactions forming the topological structure of the CCS are part of the same biological context of a CCS as proteins group within networks according to their biological function [25]. Therefore, the function of proteins in a CCS complement each other in a way that the CCS itself represents a module which is biological coherent and meaningful.

Based on those observations, we devised an algorithm which infers GO-terms based on the annotations of proteins in the same CCS for proteins with missing annotations. All CCSs with at least three interactions and a high functional similarity are considered as candidates. For qualifying CCSs we determined orthologous protein groups whose function differ significantly (p-value ≤ 0.01) from the average similarity of the CCS (dissimilar orthologous proteins) due to missing/unknown annotations. We selected those proteins and predicted the functions annotated to their orthologous partner proteins.

### Identifying GO terms in text passages

In a second phase, we tried to confirm predicted GO annotations using a novel method for the automatic identification of GO terms in natural language text [27]. We briefly describe this approach here, see [26] for more details. In general, GO terms are often constituted by several words. In this method, the decision whether or not a particular GO term is mentioned in a fraction of text is determined by a set of features. The most important feature is the occurrence of individual words constituting GO terms. However, such evidence could as well refer to other terms. The selection of the most specific term is an important aspect to avoid loss of detail. Another feature is the proximity of all the words that form the evidence of a term's mention. This idea is based on the assumption that a long distance between individual words does not support the hypothesis that they are related in the sense of forming a GO term.

The method we propose integrates the concepts of Evidence, Specificity and Proximity as described in the following. Let *t *be a GO term and let *tok*(*t*) be a set of unique words in *t*. The Specificity of a GO term *t *in a given text is defined as the amount of information carried by the term:

(1) $$I(t)=\sum_{w\in tok(t)}-log(p(w))$$

where *p*(*w*) is the probability that a word *w *occurs in that text.

The Evidence that a GO term *t *is mentioned in a continuous stretch *z *of text (also called zone) is defined as the proportion of information carried by the words present in the zone and in the term:

(2) $$e(z,t)=\frac{I(z\cap t)}{I(t)}=\frac{\sum_{w\in tok(z)\cap tok(t)}-log(p(w))}{\sum_{w\in tok(t)}-log(p(w))}$$

The Proximity of words constituting a term *t *occurring in a text is defined as the divergence of the dispersion of the term's words in the text from the term's minimum dispersion where dispersion describes the spreading of words constituting a term *t *in a zone *z*. A set of words *W *of a term *t *mentioned in the zone *z *is defined as:

(3) *W *= *tok*(*z*) ⋂ *tok*(*t*) = {*w*_0_,..., *w*_*n *- 1_}.

The dispersion of those words *W *in the zone *z *is given by the sum of distances (*dist*(*w*_*i*_, *w*_*j*_, *z*)) between a pair of words *w*_*i*_, *w*_*j *_of *W *in *z*:

(4) $$D(W,z)=\sum_{w_{i}\in W,w_{j}\in W}dist(w_{i},w_{j},z).$$

The minimum dispersion of the words *D*_*min*_(*W*) is computed as follows:

(5) $$D_{min}(W)=\sum_{i=0}^{n-1}\sum_{j=0}^{n-1}\left|i-j\right|.$$

Finally, the Proximity is given by:

(6) $$pr(W,z)=\frac{D_{min}(W)}{D(W,z)}\leq 1.$$

Evidence, Specificity and Proximity are the three parameters that are combined to score the likelihood that a term *t *is mentioned in a text zone *z*. In this work, we defined a zone *z *as a sentence. As final score, we use a weighted product:

(7) *s*(*z*, *t*) = *e*(*z*, *t*)^*α*^·*I*(*t*)^*β*^·*pr*(*W*, *z*)^*γ*^.

The parameters can be learned from annotated text. We used the second Biocreative corpus.

### Validation of GO annotations from CCSs through literature analysis

Scientific articles are primary data that contain the conceptualization of facts provided by the author from his real world perception. Human curators take this primary data source and interpret it to identify and extract protein annotations [3]. There is no solution available that automates the manual curation process and that generates results comparable to the work of a curator. Furthermore, few is yet known on how a curator derives from the text the evidence for the concepts that represent the functional annotation of a protein.

For our PPI network comparisons we considered the species *H. sapiens *(HS), *M. musculus *(MM), *S. cerevisiae *(SC) and *D. melanogaster *(DM) to identify conserved and connected subgraphs. We selected the comparisons of HS-DM, HS-SC, HS-MM and DM-SC as input data to predict and validate GO annotations against the scientific literature. Although biomedical research groups tend to focus their research on a single species and thus generate annotations for proteins of a particular organism, often their results are reported in a species independent way and due to relationships between species their research tends to be meaningful for studies in other species as well.

**Figure 2:**
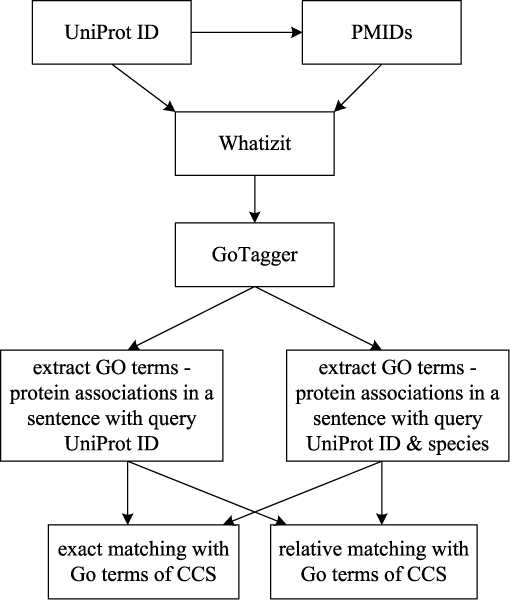
**Schematic illustration for comparing GO annotations in this study**. The flowchart summarizes all four combinations for comparing GO annotations of different resources. Protein – annotation associations were extracted from text with or without species identification and GO annotations from text and the UniProtKb/Swiss-Prot database were compared based on exact versus relative matching.

For each protein under consideration we retrieved abstracts from EBIs Medline distribution (provided from the National Library of Medicine (NLM), Bethesda, U.S.A. Last release date: 04/06/2007). All abstracts are indexed based on IDs from UniProtKb/Swiss-Prot [27]. For the indexing all names and synonyms from UniProtKb/Swiss-Prot [28] have been considered and ambiguous terms have been disambiguated using the methods described by Rebholz-Schuhmann *et al*. [27]. Retrieval of Medline abstracts has been performed using the Whatizit retrieval engine for Medline abstracts which is also accessible from outside of the EBI [29]. Furthermore, we enriched this retrieval by additional references that are contained in the BioLexicon (work in progress). The BioLexicon is a new data resource that combines the protein term repository called "BioThesaurus" with other terminological resources (e.g., NCBI taxonomy, ChEBI) and adds linguistically relevant information [30]. We used terms from the BioLexicon to retrieve additional references to Medline abstracts. All retrieved abstracts were processed and contained GO terms were automatically annotated in the document as described before (see Figure 2). Note that a given zone (e.g., a sentence) in the text can be annotated with several GO terms.

As a result of the text processing, every sentence was enriched with several annotations: (1) all GO terms that have been identified in a particular zone, (2) annotations of terms with protein UniProtKb IDs and (3) species names (tagged by a species tagger). We associated a given protein with a GO term when protein, GO term and species name were identified in the same sentence. However, requiring that the species of the protein is mentioned in the same sentence is a strong restriction, since any UniProtKb ID itself encodes already its species. Therefore, we also extracted pairs consisting of the GO term associated to a certain UniProt ID without considering the species.

We compared all identified GO terms to our predicted annotations of the proteins from the CCSs and to the annotations from other resources such as protein databases. For the comparison we use exact matching of GO terms and relative matching, where in the case of the relative matching we counted a GO term (e.g., from Medline) as a match if the term was identical to or was a direct ancestor or descendant of the term from the other sources (e.g., from CCS). Based on the relative matching we take into account the fact that GO differs in its granularity in the different branches of GO and that the specificity of terms differs across GO. Therefore, direct neighbors of a predicted term are considered to be valid and still highly specific for the annotation. Note that considering only direct neighbors is a rather strict criterion (see e.g. supplementary material of [31] for a less strict criteria).

We further studied all four combinations of the following two methods to assess annotations against literature (see Figure 2): (1) extraction of GO annotations of proteins with or without identification of the species and (2) comparison of GO annotations between resources based on exact versus relative matching.

Furthermore, we distinguished different sets of proteins according to the way how we derived GO annotations from the CCS data. The first set (**Set 1: Annotation by Orthology**) is composed of 1000 proteins that have been randomly selected from the sets of orthologous proteins identified in the first step of the algorithm. This set has been chosen to serve as a baseline for the validation of predicted GO terms.

In the second set (**Set 2: Annotations of Proteins contained in CCSs**) we included only proteins that are contained in a CCS which is the result of a pair-wise comparison between species. Proteins of this set are thus involved in interologs. We therein used species pairs HS-DM, HS-SC, HS-MM and DM-SC. The third set imposes more restrictions on the selection of proteins than Set 2 and forms a true subset of Set 2. In this set (**Set 3: Annotations and Predictions of Proteins contained in CCSs with high functional coherence**) we also considered protein annotations from the UniProtKb/Swiss-Prot database for the selection of proteins. Taking these annotations into account allows to select CCSs and proteins with high functional similarity. Set 3 can be divided into two subsets:

1. Subset **3a **includes hypothetical new GO annotations that have been transferred between orthologous proteins as described in the method section.

2. Subset **3b **comprises all protein annotations available in UniProtKb/Swiss-Prot of proteins contained in highly conserved CCSs.

**Figure 3:**
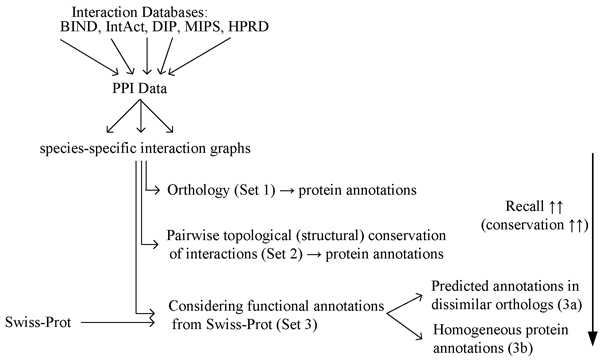
**Schematic overview of the studied protein annotations**. Schematic overview of the different sets of protein – annotation associations considered in this study.

We expect an increasing recall from the first to the last set due to an increase in biological conservation (see Figure 3). The reason is that on the one hand the number of selected proteins and annotations is going down due to stricter selection conditions. Otherwise we expect to find more proteins and their annotations in text because of their higher biological relevance in several species.

### Evaluation of extracted results

In information extraction precision and recall are defined as follows.

• Precision: Percentage of correct findings amongst all findings by a method (= 100 * true positives/(true positives + false positives)).

• Recall: Percentage of facts correctly identified by a method amongst all facts mentioned in the text. (= 100 * true positives/(true positives + false negatives))

These definitions are suitable to evaluate the performance of an analytical method on individual instances of terms and facts in the text. The gold standard is defined by the content of databases where proteins are annotated with ontological concepts by curators. This implies, that new findings are counted as false positives. Therefore, the precision of the automatically generated annotation (predicted annotation) can only be assessed from one or several curators, since they form the gold standard for the content in the public databases.

## Results

The relative matching approach was used for all proteins and their annotations from Set 1 and 2. For these sets the known GO annotations from UniProtKb/Swiss-Prot were compared with the extracted GO annotations from the literature. For proteins from Set 3 all annotations were validated by using all four combinations of the extraction methods as explained above (Figure 2).

The recall figures presented in the following are referring to annotations of a protein that have been verified by at least one occurrence of an annotation in the literature. This means that we distinguish between a validated and a non-validated annotation by counting each evidence for an association between a given protein and the mention of a GO annotation only once.

### Validation of predictions for Set 1, 2 and 3 based on annotations from the literature

Table 1 shows the results for proteins from Set 1. Annotations of orthologs have been confirmed for fly in 23% of the cases, for mouse in 27%, for human in 52% and for yeast in 55% resulting in an average recall of ~44% across all species. The low outcome for fly is the result of a low number of retrieved abstracts for fly proteins and protein functions of mouse are sparsely covered in literature, resulting in such a low recall (see Discussion).

**Table 1:** Evidences for protein – GO annotation associations in text for Set 1. Evidences in the literature for annotations of randomly chosen orthologous proteins – Set 1 – compared with relative matching.

Species	Recall
DM	470/2046 (23.0%)
MM	859/3141 (27.3%)
SC	2747/4974 (55.2%)
HS	2801/5419 (51.7%)

Table 2 lists the number of confirmed annotations of proteins from Set 2, i.e. the use of CCSs to identify conservation in the structure of subnetworks yet without consideration of functional similarities. The average recall of annotations for topological conserved proteins is now increased to ~51% in comparison to the previous set. This increase indicates that more annotations for proteins constituting interologs are represented in the literature. Similar to Table 1 the recall involving fly (especially for HS-DM) is lower compared to the rest. For DM-SC the lower results seem to be equalized by a higher accuracy of yeast.

**Table 2:** Evidences for protein – GO annotation associations in text for Set 2. Evidences in the literature for annotations from UniProtKb/Swiss-Prot considering only proteins of structurally conserved subgraphs – Set 2 – compared with relative matching.

PPI Comparison	Recall
DM-SC	34/78 (43.6%)
HS-DM	149/427 (35.0%)
HS-SC	1002/1796 (56.0%)
HS-MM	3083/6119 (49.6%)

Table 3 shows the recall of predicted and known GO annotations of Set 3. The number of confirmed GO annotations is the lowest when species matching and exact matching have been used and is highest when relative matching is used without requiring the species in the same sentence. When considering only the known annotations the average recall for any of the used methods on Set 3b is higher than the previously reported average recall for proteins from Set 1 and Set 2. In contrast to the results in Table 1 and 2 the recall for the known annotations with relative matching improved significantly for Set 3b up to ~82%.

**Table 3:** Evidences for protein – GO annotation associations in text for Set 3. Comparing newly predicted GO terms (Set 3a) and known GO terms (Set 3b) from UniProtKb/Swiss-Prot with protein – GO annotation associations in Medline using different extraction criteria.

Extraction criteria	GO term Set	Recall
Exact & Species	predicted GO terms	19/88 (22%)
	known GO terms	129/283 (46%)
Relative & Species	predicted GO terms	21/88 (24%)
	known GO terms	164/283 (58%)
Exact	predicted GO terms	31/88 (35%)
	known GO terms	201/283 (71%)
Relative	new GO terms	34/88 (39%)
	known GO terms	234/283 (82%)

For the results of the relative matching method of Set 2 and 3 we also determined the redundancy of evidences in Medline. By redundancy, we mean the total number of occurrences of a specific protein – GO term association in Medline abstracts (which are represented in the recall as one hit). In detail, we gathered for Set 2 and 3 the median, the maximum and the average frequency of protein – GO term associations in text summarized in Table 4. Furthermore, we specified those frequencies separately for the three ontologies. We computed a median frequency of 11 for Set 2, and 33 and 9 respectively for Set 3 considering known and predicted annotations separately. Overall the highest frequencies have been observed for the subontology molecular function (MF).

**Table 4:** Redundancy of protein – GO term associations in Medline. Median, maximum and average frequencies of protein – GO term associations for proteins of Set 2 and 3 in Medline.

Protein Set	Frequencies	Total	MF	BP	CC
Set 2	median	11	15	10	7
	max.	19855	2990	19855	3132
	mean	72	77	89	25
Set 3a (predicted)	median	9	12	18	3
	max.	907	907	88	66
	mean	47	55	40	3
Set 3b (known)	median	33	51	18	27
	max.	6566	6566	2053	1823
	mean	199	328	153	87

Similar observations have been made when considering the distribution of the detected terms across the three branches of GO in Set 3. Table 5 shows the recall of known GO terms measured for the three subontologies of GO. Again the recall increased when moving from exact matching to relative matching and moving away from considering the species increased the recall again. Recall was lowest for biological process (BP) and was higher for cellular component (CC) and highest for molecular function (MF). Regarding the identification of GO terms in text other research shows that cellular location can be more precisely identified than for example molecular function [32,33].

**Table 5:** Distribution of confirmed GO terms across the three subontologies of GO. Subontology specific consideration of known GO terms (Set 3b) confirmed by literature.

Extraction criteria	Recall – MF	Recall – BP	Recall – CC
Exact & Species	56/107 (52%)	31/85 (36%)	42/91 (46%)
Relative & Species	71/107 (66%)	41/85 (48%)	52/91 (57%)
Exact	83/107 (77%)	51/85 (60%)	67/91 (73%)
Relative	90/107 (84%)	69/85 (81%)	75/91 (82%)

The GoTagger tags a sentence with a list of GO terms occurring within this sentence sorted by the significance and evidence of the GO terms. The distribution of the confirmed GO terms of Set 3 (predicted and known) across this list was considered as well as observed in which position of the list they were placed. The list was divided into blocks of positions 1 – 5, 6 – 10, 11 – 20 and > 20. The occurrences of predicted and known GO terms in these ranges are shown in Table 6. Except for the first row in the table of the predicted terms the majority of the confirmed GO terms is always in first part of the list, and thus belongs to the more significant terms in the text which shows that our GO tagging method is reasonable.

**Table 6:** Distribution of confirmed GO terms by significance and evidence. Distribution of the identified terms over the list specified by the GoTagger separated into predicted (3a) and known annotations (3b).

	predicted GO terms					known GO terms				
Extraction criteria	# terms	1–5	6–10	11–20	>20	# terms	1–5	6–10	11–20	>20
Exact & Species	19	5	6	3	5	129	60	35	16	18
Relative & Species	21	10	8	2	1	164	82	37	20	25
Exact	31	18	7	3	3	201	125	39	22	15
Relative	34	25	5	3	1	234	163	36	17	18

### Prediction precision

Set 3a comprised in total 88 predicted GO annotations. Out of this set 34 GO annotations (see supplementary Table S7 in Additional file 1) were confirmed from the literature based on relative matching. To estimate the accuracy of these predicted annotations the findings were given to a GO curator for assessment.

All predictions were found to be correct (100% precision) and all have been verified through evidence gathered from the electronic data resources available to the GO curators. Be reminded that all 34 annotations are not yet contained in UniProtKb/Swiss-Prot (but will be soon).

15 predicted GO annotations were verified through evidence retrieved in QuickGO and thus were not completely new annotations for the respective proteins. This discrepancy can be explained by the fact that we used only manually curated annotations as a basis for functional assessment and function prediction. Annotations from QuickGO confirming the predicted GO terms are inferred by electronic annotation using automatically methods such as Interpro or UniProt Enzyme Code mapping. This means that these 15 GO annotations were approved by other automatic prediction approaches and that they would still require manual annotation. The combination of our methods could be used to filter out such annotations for automatic integration into UniProtKb/Swiss-Prot.

The remaining GO annotations in our set accounted for truly novel GO annotations for the considered proteins. Those terms differed in their specificity. Beside a few unspecific annotations, such as *protein binding or transcriptional activator activity*, the majority of proposed terms was rather specific, e.g. *interleukin-7 receptor activity, α-glucosidase II complex and histone acetyltransferase activity*. Validation of the proposed protein functions was approved by the CC lines of the corresponding UniProtKb/Swiss-Prot entries which are given in the supplementary Table S8 in Additional file 1. The CC lines contain free text comments on an entry to convey additional relevant information for a protein. Those comments are categorized into different topics according to the information they cover and especially the topics function, subunit or subcellular location yielded information to verify our predictions. Within these comments implicit protein information about function, biological process or cellular location are represented.

However, these information have not been used for annotating the proteins explicitly with the corresponding GO terms, but those free text comments seem to be suited to identify and extract GO terms.

## Discussion

In this study, we analysed proteins and annotations of publicly available PPI data from several species. Considering the species separately, yeast proteins and their functions seem to be most frequently described in Medline. This is not surprising to us because yeast is known as one of the main model organism in different research areas. Surprisingly, we achieved the lowest recall of confirmed GO terms for fly proteins.

We expected annotations of fly proteins to be found more frequently in literature because *D. melanogaster *is also a common model organism as yeast. The low recall in this analysis is probably strongly influenced by the difficulties in retrieving abstracts for the majority of fly proteins indicating the challenge of recognizing protein names of *D. melanogaster *in literature [13]. Therefore, it was problematic to link GO terms to protein IDs in text, and consequently a smaller number GO terms have been extracted and compared. The recall of mouse and human protein functions lies in-between yeast and fly, where human protein functions are more frequent described and the recall is close to the one of yeast. The recall of mouse is similar to the recall of fly but slightly higher. Since mouse and human are very close we expected a similar recall for both species, but human proteins and their functions are more frequently discussed than mouse protein functions. Proteins of human seem to be more often subject of detailed small-scale studies because of their increased relevance in the onset of diseases and their functions are therefore more frequently covered in the scientific literature what accounts for the difference between the recalls.

When considering only protein sets with known annotations, the average recall for annotations validated by published evidences increased starting from orthologous proteins (Set 1, ~44%) to proteins involved in conserved interactions (Set 2, ~51%) to proteins showing not only topological but also a strong functional conservation (Set 3b – known, ~82%). The increase of the recall in these three sets correlates to the level of biological conservation of the involved proteins and interologs (see Figure 3). This correlation indicates that proteins that are strongly conserved across various species are often well-studied and thus are described more explicitly in literature. This phenomenon could be called conservation of facts similar to the conservation of protein functions in CCSs.

We considered 3a as a separate set, since it contains only predicted GO annotations in contrast to Set 1, 2 and 3b. The recall for predicted terms (3a) in Medline is 39%, half of the recall for known terms obtained from UniProtKb/Swiss-Prot (82%). This means that ~40% of the predicted annotations are described in the literature but are not yet contained in UniProtKb/Swiss-Prot. The question is why the remaining 60% of the predicted annotations are not contained in the literature. 18% of the known annotations are not found in the current form in Medline abstracts. Those annotations are curated information from literature and should be found in biomedical text. This could be explained by the fact that the GO terminology is not designed for text mining and therefore the undetected GO terms are difficult to identify with the current techniques. This probably accounts also for the number of unconfirmed predicted annotations since the annotations from UniprotKb/Swiss-Prot provide the basis for function prediction. Another reason is the inhomogeneous distribution of GO terms within Medline, i.e. there are GO terms more frequently described in abstracts and others mentioned less frequently or not at all. Furthermore, until now we considered only annotations contained in abstracts. We expect different results when applying the known techniques to full papers.

Our method is not optimized to predict large amounts of novel protein functions, but for achieving a very high precision level. This emphasises the importance of combining different prediction approaches and shows that our method and results are applicable for protein function prediction.

## Conclusion

In our approach for automatic annotation of proteins with gene ontology terms we combine two distinct methods, i.e. the generation of connected and conserved subgraphs (CCSs) from PPI graphs with literature mining. To our knowledge such an approach has not been followed up to now. Applying these methods we achieved predictions at a high precision level outperforming other methods.

This excellent result is mainly due to the fact that the molecular function of conserved proteins is well known to researchers even if the annotation is not yet contained in the public scientific databases. This interpretation is supported from our finding that the predicted annotations were found in the scientific literature for verification. Finally, a trained GO curator confirmed that all our newly predicted GO annotations which were approved by literature were correct. This result suggests that conserved proteins and their functions are explicitly discussed in the literature, i.e. these facts could be called conserved facts from the literature.

Our approach shows that both data resources, databases and literature coalesce regarding their information content which is necessary for a reliable resourcing of biological data and in case of protein annotations for a sufficient coverage of proteins with GO terms. Our findings should encourage other scientists to increasingly rely on automatic prediction methods that integrate different resources including literature to generate reliable results based on automatic processing techniques.

## Competing interests

The authors declare that they have no competing interests.

## Authors' contributions

SJ: developed the method to identify conserved protein interaction subgraphs and to predict missing protein functions, carried out the studies described in this paper and contributed to the manuscript. SG: developed and implemented the text mining method for identifying GO terms in text and contributed to the manuscript. UL: conceived part of the study and contributed to the manuscript. DR: conceived part of the study and contributed to the manuscript. All authors read and approved the final manuscript.

## Supplementary Material

Additional file 1 **Predicted GO annotations and supporting evidences**. List of predicted and confirmed GO terms and additional information that support their correctness. This file contains the two supplementary tables S7 and S8. Table S7 presents the set of 34 predicted GO terms that were confirmed from the literature based on relative matching and found to be correct by manual assessment by a GO curator. Table S8 lists truly novel GO terms that were not predicted by other automated function prediction methods as well as the corresponding evidences from the UniProtKb/Swiss-Prot CC lines that support their correctness. Click here for file
