# Supplementary material for: Integrating protein-protein interactions and text mining for protein function prediction
Source: BMC Bioinformatics. 2008 Jul 22;9(Suppl 8):S2. doi: 10.1186/1471-2105-9-S8-S2 (PMC2500093; doi:10.1186/1471-2105-9-S8-S2)
Supplement: Additional file 1 — Predicted GO annotations and supporting evidences. List of predicted and confirmed GO terms and additional information that support their correctness. This file contains the two supplementary tables S7 and S8. Table S7 presents the set of 34 predicted GO terms that were confirmed from the literature based on relative matching and found to be correct by manual assessment by a GO curator. Table S8 lists truly novel GO terms that were not predicted by other automated function prediction methods as well as the corresponding evidences from the UniProtKb/Swiss-Prot CC lines that support their correctness. [file 1471-2105-9-S8-S2-S1.pdf]

Supplementary Material for: Integrating Protein-Protein Interactions and Text Mining for Protein Function Prediction

Samira Jaeger [1,2], Sylvain Gaudan [2], Ulf Leser [1], Dietrich Rebholz-Schuhmann [2]

[1]Knowledge Management in Bioinformatics, Humboldt-University Berlin, Unter den Linden 6, 10099 Berlin, Germany
[2]European Bioinformatics Institute, Wellcome Trust Genome Campus, Hinxton, Cambridge, CB10 1SD, UK

Table 7: List of predicted GO annotations that were confirmed from the literature based on relative matching on the one hand and found to be correct by manual assessment by a GO curator otherwise.

UniProt ID	GO ID	GO Term
P10964	GO:0005515	Protein binding
P08518	GO:0003677	DNA binding
P62487	GO:0003899	DNA-directed RNA Pol
P34087	GO:0005515	Protein binding
P62487	GO:0006366	Transcription from RNA Pol II promotor
P53803	GO:0003899	DNA-directed RNA Pol
P40422	GO:0008270	Zinc ion binding
Q9UBU8	GO:0004402	Histone acetyltransferase activity
Q9UBU8	GO:0005515	Protein binding
P07703	GO:0006360	Transcription from RNA Pol I promotor
P32910	GO:0006359	Regulation of transcription from RNA Pol III promoter
P38431	GO:0003743	Translation initiation factor activity
P38431	GO:0003924	GTPase activity
P38431	GO:0005515	Protein binding
P32502	GO:0005085	Guanyl-nucleotide release factor activity
P05198	GO:0006413	Translation initiation
O75821	GO:0005852	Eukaryotic translation initiation factor 3 complex
Q02748	GO:0005515	Protein binding
O44437	GO:0005515	Protein binding
P34902	GO:0004917	Interleukin-7 receptor activity
P34902	GO:0004913	Interleukin-4 receptor activity
P34902	GO:0004911	Interleukin-2 receptor activity
P34902	GO:0006955	Immune response
P14314	GO:0004558	α-glucosidase activity
P14314	GO:0005515	Protein binding
Q9QXK9	GO:0005737	Cytoplasm
Q14697	GO:0017177	α-glucosidase II complex
Q9Y618	GO:0003677	DNA binding
Q9Y618	GO:0016564	Transcriptional repressor activity
Q9WU42	GO:0003714	Transcription corepressor activity
Q9WU42	GO:0005112	Notch binding
Q14814	GO:0005634	Nucleus
P10242	GO:0003700	Transcription factor activity
P06876	GO:0016563	Transcriptional activator activity

Table 8: Truly novel GO annotations and their support information to validate predictions found in CC lines of the respective UniProt entries.

UniProt ID	GO ID	Free text comments to support predictions
P10964	GO:0005515	SUBUNIT: Component of the RNA polymerase I (Pol I) complex consisting of 14 subunits.
P34087	GO:0005515	SUBUNIT: Component of the RNA polymerase II (Pol II) complex consisting of 12 subunits. RPB4 and RPB7 form a subcomplex that protrudes from the 10-subunit Pol II core complex.
Q9UBU8	GO:0004402	FUNCTION: Component of the NuA4 histone acetyltransferase (HAT) complex which is involved in transcriptional activation of select genes principally by acetylation of nucleosomal histone H4 and H2A.
P07703	GO:0006360	FUNCTION: DNA-dependent RNA polymerase catalyzes the transcription of DNA into RNA using the four ribonucleoside triphosphates as substrates.
P32910	GO:0006359	SUBUNIT: Component of the RNA polymerase III (Pol III) complex consisting of 17 subunits.
P38431	GO:0003924	FUNCTION: Catalyzes the hydrolysis of GTP bound to the 40S ribosomal initiation complex (40S.mRNA.Met-tRNA[F].eIF-2.GTP) with the subsequent joining of a 60S ribosomal subunit resulting in the release of eIF-2 and the guanine nucleotide.
P38431	GO:0005515	SUBUNIT: Monomer. Interacts with NIP1 and SUI3.
P34902	GO:0004917	SUBUNIT: The gamma chain is common to the IL2, IL4, IL7, IL21 and probably also the IL13 receptors.
P34902	GO:0004913	SUBUNIT: The gamma chain is common to the IL2, IL4, IL7, IL21 and probably also the IL13 receptors.
P34902	GO:0004911	SUBUNIT: The gamma chain is common to the IL2, IL4, IL7, IL21 and probably also the IL13 receptors.
Q9QXK9	GO:0005737	SUBCELLULAR LOCATION: Cytoplasm.
Q14697	GO:0017177	SUBUNIT: Heterodimer of a catalytic alpha subunit (GANAB) and a beta subunit (PRKCSH)
P06876	GO:0016563	FUNCTION: Transcriptional activator;
P34902	GO:0006955	FUNCTION: Common subunit for the receptors for a variety of interleukins.
O44437	GO:0005515	from abstract of PMID: 17105994
